# A low-cost, portable, dual-function readout device for amplification-based point-of-need diagnostics

**DOI:** 10.1128/aem.00902-23

**Published:** 2023-12-04

**Authors:** Yiping Zou, Michael Glenn Mason, Jose Ramon Botella

**Affiliations:** 1 College of Life Sciences, Nanjing Agricultural University, Nanjing, Jiangsu, China; 2 School of Agriculture and Food Sciences, The University of Queensland, Brisbane, Queensland, Australia; Danmarks Tekniske Universitet, The Novo Nordisk Foundation Center for Biosustainability, Lyngby-taarbæk, Denmark

**Keywords:** point-of-need diagnostics, isothermal amplification, readout device, inexpensive, portable

## Abstract

Amplification-based diagnostics has been widely used in plant disease diagnostics due to its sensitivity, rapidness, and flexibility. The modules to interpret amplification results rapidly, reliably, and easily in resource-limited settings (e.g., field-based environments) are in high demand. Therefore, we have designed and developed a simple, low-cost, easy-to-perform, and portable device named Dr. Diagnose to aid accurate, unbiased, and user-friendly readout of amplification reactions outside the model laboratory environment. Dr. Diagnose is an Arduino microcontroller-based light meter that can operate with a portable power bank. It allows the user to select to analyze either fluorescence or turbidity levels in amplification reactions by simply touching the button and informs the user of whether the reaction’s fluorescence or turbidity exceeds a set threshold and is thus considered positive within seconds. The sensitivity of Dr. Diagnose was found to be comparable to gel electrophoresis-based detection. Dr. Diagnose has a simple circuit containing only 13 electronic components, which means it can be easy to generate, affordable, and accessible to the broader community. The development of Dr. Diagnose provides a practical and inexpensive readout tool for amplifications at point-of-need in a rapid, accurate, and user-friendly manner.

**IMPORTANCE:**

The first critical step in timely disease management is rapid disease identification, which is ideally on-site detection. Of all the technologies available for disease identification, nucleic acid amplification-based diagnostics are often used due to their specificity, sensitivity, adaptability, and speed. However, the modules to interpret amplification results rapidly, reliably, and easily in resource-limited settings at point-of-need (PON) are in high demand. Therefore, we developed a portable, low-cost, and easy-to-perform device that can be used for amplification readout at PON to enable rapid yet reliable disease identification by users with minimal training.

## INTRODUCTION

Nucleic acid amplification-based technologies have been widely used in plant disease diagnostics (1, 2). Current nucleic acid amplification-based analysis typically involves complex methodologies and thus requires well-trained personnel with access to a variety of sophisticated equipment in a laboratory, which makes them impractical to perform in resource-limited settings (e.g., field-based environments) (3). These constraints result in increased costs incurred by sample transportation as well as delays in disease identification and management (4).

Rapid and simple nucleic acid purification methods have been developed for point-of-need (PON) purposes, making it possible to process samples outside a modern laboratory. Alkaline polyethylene glycol lysis buffer and a handheld tissue homogenizer have successfully been used to extract crude DNA from various plants for amplification without the aid of laboratory equipment (5, 6). Nucleic acid purification dipstick technology utilizes cellulose-based dipstick to purify DNA or RNA from various biological samples in less than 30 seconds without any electrical equipment or pipetting steps (7–9).

Isothermal amplification is a powerful technology for PON applications since it can be performed at one single constant temperature provided by a simple heat source (e.g., water bath) (10). Loop-mediated isothermal amplification (LAMP) is an ideal isothermal amplification method for PON applications (11). The Bst DNA polymerase used in LAMP has intrinsic reverse transcriptase activity (12), making it easy to use LAMP for both isothermal DNA and RNA detection. Low-cost chemical heaters have been developed for LAMP, which utilize the heat produced from exothermic chemical reactions (e.g., calcium oxide and water, magnesium iron ally and water) (13–15). Successful LAMP amplifications have also been performed using the commonly available disposable hand warmers, a chemical heater, when the environment temperature was between 4°C and 37°C (16, 17). Another research group created a viable isothermal incubator by mixing boiling water with cold water in a coffee thermos to obtain the desired reaction temperature (18). The commercial travel coffee mug for cars can be used as the incubator for LAMP as well since it maintains a temperature of 65°C, which is in the range of LAMP incubation temperature. Therefore, a number of viable means can create a portable, low-cost incubator suitable for LAMP outside modern laboratory at PON.

Several readout methodologies have been developed to identify successful LAMP amplification. Lateral flow technology has been reported by a number of research groups as a viable option for PON applications (19). In this technology, labeled LAMP amplicons are transferred to a lateral flow strip and allowed to flow across the strip, and if present, the amplicons are immobilized on the test line turning it red due to the presence of gold nanoparticles (6, 20, 21). Color-change indicators that do not inhibit amplification have been incorporated into LAMP reactions to enable the detection of amplification without the need to transfer LAMP amplicons to a lateral flow strip. The addition of hydroxynaphthol blue (HNB) and pH-sensitive dyes (e.g., cresol red and neutral red) prior to amplification induces a color change in positive amplifications discernible by the naked eye (22–24). However, the color change induced by HNB is not always sharp enough for the naked eye (24). pH dye-based naked-eye detections typically induce a clear color change (24), but they require a neutral nucleic acid sample. This requirement is not well-suitable for plant samples processed by PON nucleic acid purification methods since pH varies among different plant species (25) or the buffer used is not neutral (5). Finally, the presence of LAMP-based amplicons can be detected using readouts based on flocculation using different nanoparticles (26, 27). Although fast and inexpensive, this method needs to add the flocculation reagents after the reaction is finished, opening the amplification tubes and increasing the probability of cross-contamination.

The simplest and cheapest readout method is based on turbidity (cloudiness) caused by the generation of insoluble by-products (i.e., magnesium pyrophosphate) in successful LAMP reactions (28, 29). The turbidity-based readout is advantageous for PON applications as it does not require any additional equipment or reagents. However, variable light conditions (e.g., sunlight) can affect the user’s ability to identify turbid reactions by the naked eye and potentially result in incorrect interpretation of the results (23, 30).

Overall, the combination of simple nucleic acid purification methods (5, 7) and isothermal amplification technology makes it possible to perform amplification by users with minimal training outside the modern laboratory environment. However, the modules to interpret amplification results rapidly, reliably, easily, and user-friendly in resource-limited settings are in high demand. Therefore, we have developed a hand-held, battery-powered, inexpensive device to enable unbiased and accurate measurements. By simply touching a button, the device automatically analyzes the turbidity/fluorescence of amplification reactions and outputs interpreted results (positive/negative) to an LCD screen, providing a practical tool as the amplification readout for PON applications.

## RESULTS

### Development of a turbidity-based DNA amplification readout

To develop an electronic tool capable of measuring the development of turbidity in LAMP amplification, we built a basic electronic circuit based on an Arduino UNO microcontroller (https://www.arduino.cc/en/Guide/Introduction) controlling a light source circuit and a sensor circuit (Fig. S1). In the light source circuit, a blue light-emitting diode (LED) with an emission peak at 450 nm was used to illuminate the sample. The intensity of the light source was controlled by placing the LED in line with a potentiometer (i.e., variable resistor). The sensor circuit was built to measure the relative intensity of the light that either passed through a sample or was scattered by insoluble particulates in the sample using a phototransistor in line with a potentiometer. Light perception by the phototransistor induced a current in the circuit and thus produced a change in voltage across the potentiometer, which was measured by an Arduino’s analog-to-digital-converter (ADC) to provide a relative light intensity value (i.e., sensor reading) for the sample.

**Fig 1:**
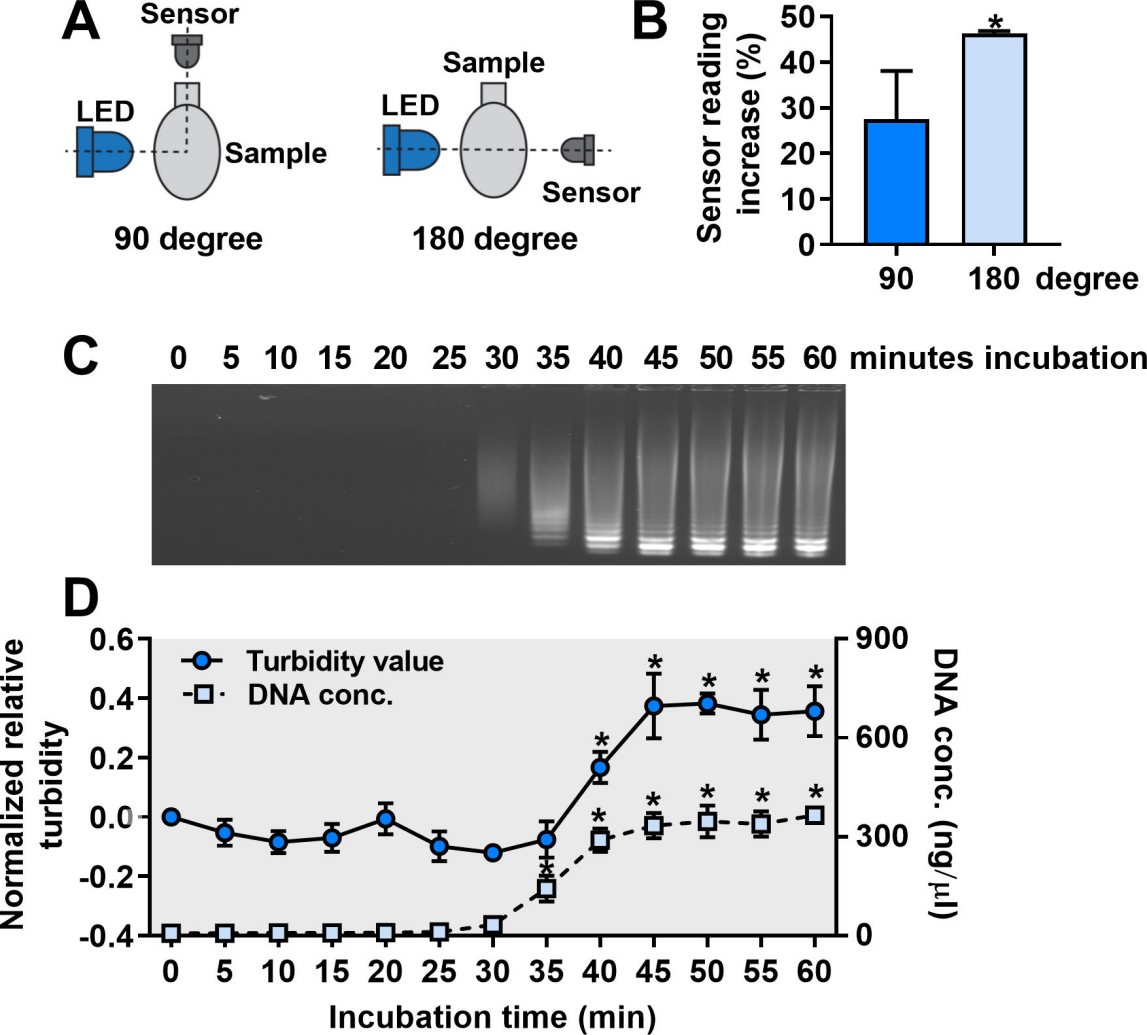
Optimization of turbidity measurements. (**A**) The light sensor was placed at an angle of either 90° or 180° to the LED (top view). (**B**) Sensor reading increases between negative and positive samples were calculated and plotted. Statistically significant differences were determined using an unpaired *t* test (*n* = 3, *P* < 0.05). (**C**) Identical LAMP reactions containing 4 fg of DNA template were incubated at 63°C for up to 60 minutes and analyzed using agarose gel electrophoresis. Two additional identical sets of LAMP reactions were prepared showing similar results to those shown in Fig. 1C. (**D**) DNA concentration and normalized turbidity of reactions from three biological replicates were measured and plotted against incubation time. At each time point (*T*), normalized relative turbidity was calculated as [(light intensity at 0 minute/light intensity at time *T*) − 1]. The asterisk above data points indicates statistically significant differences in either DNA concentration or normalized relative turbidity compared with the control (0 minute) using one-way ANOVA (*n* = 3, *P* < 0.05).

Light intensity measurements were performed by placing the light sensor at an angle of either 90° or 180° relative to the LED (Fig. 1A). LAMP reactions displaying ladder-like patterns in agarose gel were used as positive samples, and reactions without visible amplicons were used as negative samples. The light sensor positioned at a 90° angle detected an increase of 28% in sensor readings of positive samples compared to negative ones, while the sensor placed at a 180° angle detected a 46% increase using the same samples (*n* = 3, *P* < 0.05) (Fig. 1B). All turbidity measurements from this point on were performed using the sensor at a 180° angle from the LED.

To prepare LAMP reactions with different turbidity levels, a series of identical LAMP reactions were incubated for 0, 5, 10, 15, 20, 25, 30, 35, 40, 45, 50, 55, or 60 minutes. Gel electrophoresis revealed evidence of amplification in the reaction at 35 minutes, which had a faint but visible LAMP amplification banding pattern corresponding to an amplicon concentration of 143 ng/µL (Fig. 1C and D). Amplicon DNA concentration increased at 40 minutes and reached a plateau at 45 minutes with little or no increase observed afterward. The turbidity value significantly increased at 40 minutes, reached a maximum at 45 minutes, and remained at similar levels thereafter (Fig. 1D). The fact that turbidity and DNA concentration values reached a plateau at the same time suggests exhaustion of amplification reagents at 45 minutes. This indicates that turbidity measurements by the initial electronic device provide a clear indication of amplification.

### Optimization of fluorescence-based DNA quantification

To measure fluorescence, a filter needs to be placed in between the light sensor and the sample to limit the amount of blue (excitation) light being received by the light sensor as well as allow green (fluorescent emission) light through. The performance of a range of low-cost color filters designed for use in photography lighting was assessed for their ability to distinguish between positive and negative LAMP reactions containing the dsDNA intercalating fluorescent dye SYTO9. In the absence of the filter, positive reactions displayed 24% higher sensor readings compared to negative controls, which was detected by the light sensor positioned at a 90° angle to the LED (Fig. S2A). Among the five filters tested, filter #2 (i.e., orange filter) and filter #5 (i.e., yellow filter) provided the highest contrast between the positive and negative samples with 61% and 66% increases in sensor readings, respectively. The remaining filters tested (filters #1, #3, and #4) did not provide satisfactory results (Fig. S2A). The yellow filter was used for all subsequent fluorescent measurements.

Optimization of the light source/sensor architecture for fluorescence measurements was performed in a similar manner to the turbidity experiments described above. Fluorescence measurements of LAMP reactions were made with the light sensor placed at an angle of 90° or 180° to a blue LED as shown in Fig. 1A. Unlike the turbidity observations, light sensors positioned at a 90° angle to the light source provided the best results with a 40% increase in sensor readings for positive reactions compared to negative controls, while sensors positioned at a 180° angle provided a 34% increase (Fig. S2B). Thus, a 90° angle between the sensor and blue LED was used for all subsequent fluorescence measurements.

To determine the optimal concentration of fluorescent dye, we tested three different concentrations of SYTO9 (1.25, 2.5, or 3.75 µM) in independent LAMP reactions. A final concentration of 2.5 µM provided the highest sensor reading difference (46%) between positive and negative LAMP reactions (Fig. S2C) and was used for the fluorescence assays for the remainder of this study.

**Fig 2:**
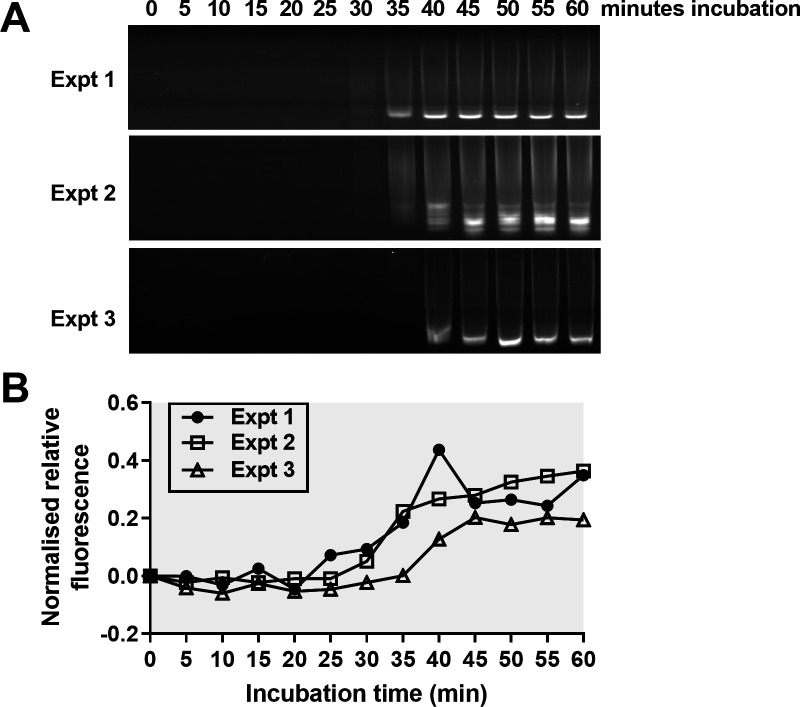
Fluorescence measurements in LAMP reactions. (**A**) Identical LAMP reactions were incubated at 63°C for up to 60 minutes and visualized in agarose gels. (**B**) Fluorescence levels of each reaction were measured with the light sensor/yellow filter placed at a 90° angle to the LED. At each time point (*T*), normalized relative fluorescence levels were calculated as [(sample reading at time *T*/sample reading at 0 minute) − 1]. Expt, experiment.

The fluorescence-based detection limit was studied by analyzing a series of identical LAMP reactions incubated for up to 60 minutes. Unlike the turbidity experiments described above, the presence of SYTO9 in these samples prevents accurate fluorimeter quantification of the amplicon DNA concentration. Thus, the amplification level in each reaction was indirectly evaluated using agarose gel electrophoresis. Our observations showed a correlation between the measured fluorescence levels and the agarose gel results. In two out of three independent experiments (Expt #1 and #2), LAMP amplification banding pattern was visible at 35 minutes (Fig. 2A) and was accompanied by an increase in fluorescence values (Fig. 2B). A third biological replicate (Expt #3) showed a similar pattern to the first two experiments in the electrophoresis gel and fluorescence measurements albeit with a delay of 5 minutes (Fig. 2).

### Design and construction of a portable DNA amplification readout device

**Fig 3:**
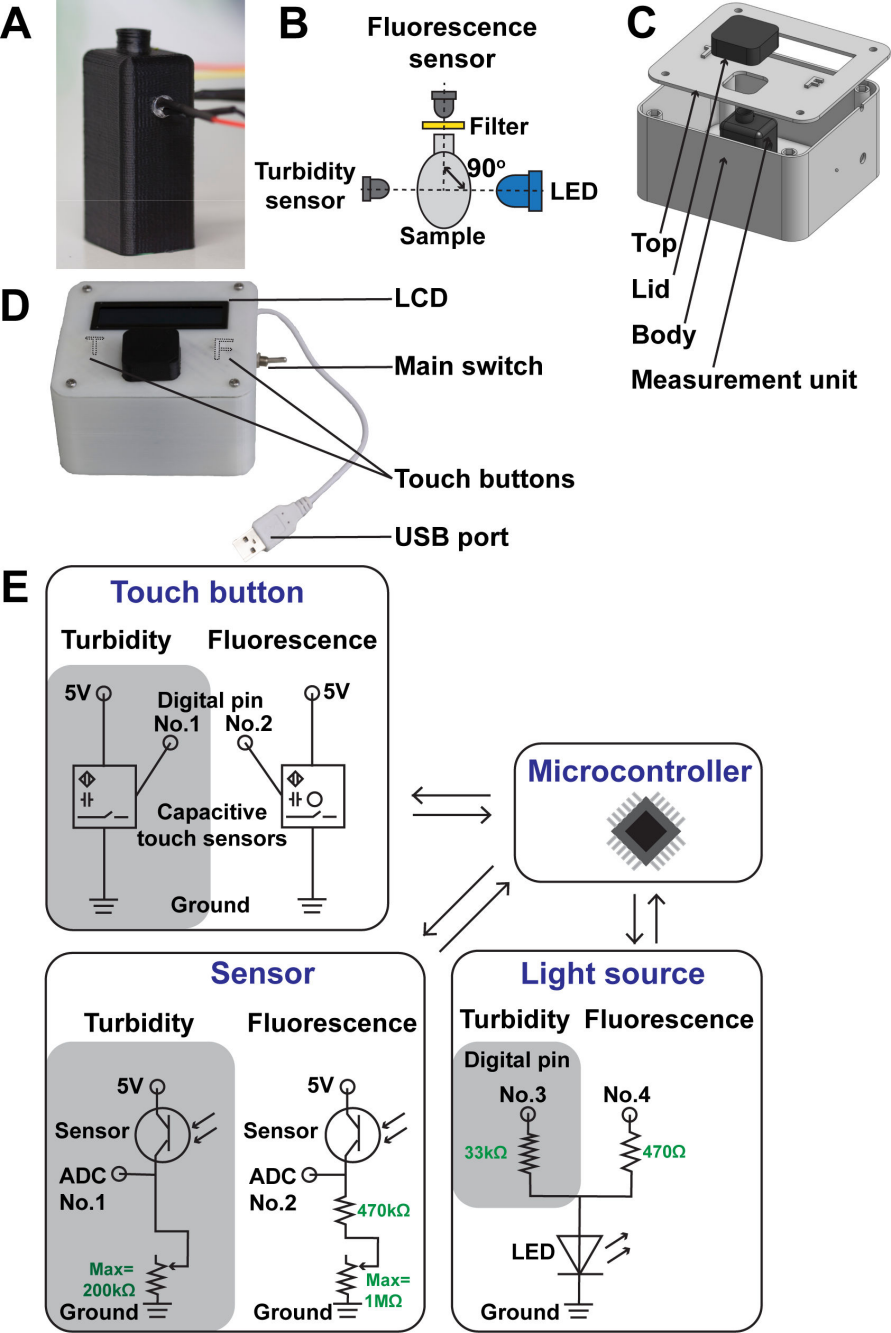
Design and construction of the portable readout device. (**A**) Photograph of the measurement unit (25 × 20 × 55 mm, L × W × H). (**B**) Top view of the different elements in the measurement unit. (**C**) 3D printed device including body, top, measurement unit, and lid. (**D**) Photograph of the device (108 × 90 × 55 mm, L × W × H). A USB plug was used to connect to a power supply such as a portable USB battery. A main switch controlled the power. “T” and “F” touch buttons were used to start the turbidity and fluorescence measurements, respectively. The LCD was used as a user interface to provide instructions and results. (**E**) Circuit of electronic components in Dr. Diagnose.

Based on the data from the experiments described above, we designed and built a device capable of simultaneously measuring turbidity and fluorescence in amplification reactions named Dr. Diagnose (Fig. 3). A measurement unit was built including a 3D printed sample holder to accommodate a blue light LED, two light sensors, and a 0.2 mL amplification tube (Fig. 3A and B). The LED was positioned at the side of the tube at a height appropriate to the level of a typical reaction volume of 25 µL. The light sensors for turbidity and fluorescence were attached to the sample holder with the turbidity sensor placed at an angle of 180° to the LED and the fluorescence sensor at a 90° angle with a yellow filter in front of it (Fig. 3B). A lid for the sample holder was designed to avoid interference from external light sources (Fig. 3C). All electronic components were connected to a prototyping board as described in the circuit shown in Fig. 3E. Capacitive touch sensors were glued in place on the underside of the device’s top plate directly beneath “T” and “F” symbols to measure the sample’s turbidity or fluorescence, respectively (Fig. 3D). A 16 × 2 character LCD display interface provides the user with instructions and displays the interpreted results (positive/negative) of each sample after measurement. A USB plug was incorporated into the circuit so that the device can be powered from commercially available USB phone power banks supporting low current charging. After powering the unit, the LCD directs the user to choose between turbidity and fluorescence measurements by touching either the “T” or “F” symbols on the device.

### Device calibration

**Fig 4:**
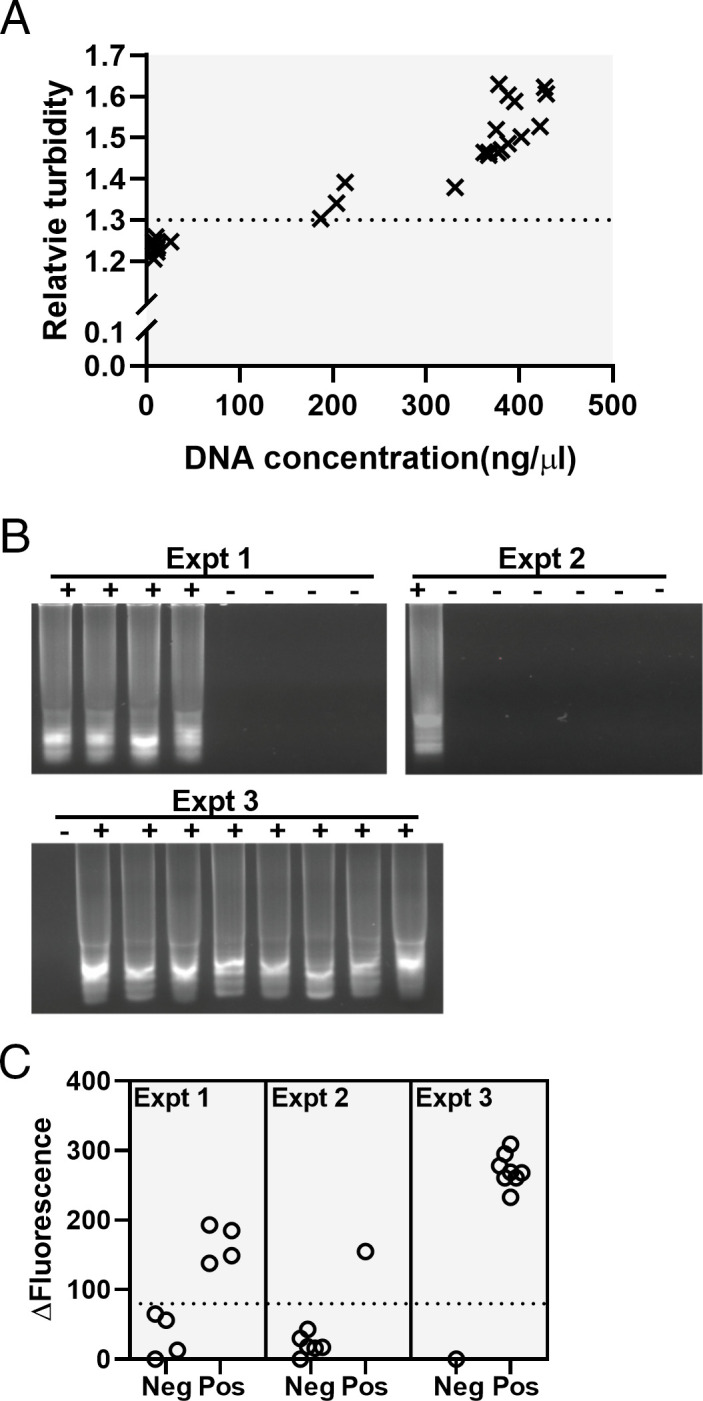
Device calibration. (**A**) The relative turbidity values of LAMP reactions were calculated by Dr. Diagnose and their DNA concentrations were measured. (**B**) LAMP reactions containing 2.5 µM SYTO9 were performed in three independent experiments, and the amplification products were analyzed using agarose gel electrophoresis. Reactions with visible amplicons in the gel were classified as positive (+), while those without visible products were classified as negative (−). (**C**) Dr. Diagnose was used to measure the fluorescence values of the reactions in Fig. 4B. For each independent experiment, the lowest fluorescence reading among the negative reactions was subtracted from all readings in the same experiment (ΔFluorescence). Pos, positive samples; Neg, negative samples; and Expt, experiment.

Device calibration was achieved by performing multiple independent experiments, each containing positive and negative LAMP reactions, and analyzing the final products of the reactions. To calibrate turbidity readings, the DNA concentration of completed reactions was measured and plotted against the relative turbidity values (i.e., sensor reading in the absence of samples/sensor reading of a sample) (Fig. 4A). A correlation was observed between the final DNA concentration and the relative turbidity with the data fitting to a regression line with an *r*^2^ value of 0.89. Based on these results, a turbidity threshold limit of 1.3 was set to identify samples considered to have sufficiently amplified a product to be considered positive.

The raw data of a commercial real-time PCR machine were not firmly fixed at the initial stage of PCR amplification (Fig. S3A), possibly because the fluorescence values of these reactions were not large enough to mask electronic noise generated by the electronic circuit (31), and/or amplicons generated fluorescence but were not visible in agarose gel. Similarly, we found that fluorescence readings measured by Dr. Diagnose varied among negative LAMP reactions although these samples did not display visible amplicons in agarose gels. To calibrate the fluorescence readings of Dr. Diagnose, ideally, we should establish a threshold value to distinguish fluorescence readings of positive LAMP reactions from the maximum fluorescence reading of negative reactions. To achieve this, three individual experiments including multiple LAMP reactions were performed. The fluorescence of the final amplification products was measured before analyzing them by gel electrophoresis (Fig. 4B). The data were analyzed by determining the lowest fluorescence reading among negative samples for each independent experiment and subtracting this value from all readings in the same experiment (Δ Fluorescence). Analysis of the Δ Fluorescence data showed obvious differences between positive and negative samples both within each experiment as well as across all experiments (Fig. 4C), revealing that a Δ Fluorescence threshold value of 80 is sufficient to distinguish between successful (positive) and unsuccessful (negative) amplifications.

Based on our data analysis, Dr. Diagnose was programed to interpret the turbidity or fluorescence of samples and display in the LCD whether the sample can be considered positive or negative. For fluorescence measurements, the user is instructed to insert a reference tube (negative control) allowing the device to measure and store the background control light intensity. Negative controls are not required in turbidity measurements. After the user is prompted to insert the sample, the device measures light intensity, performs calculations, and provides a diagnostic of positive or negative, depending on the calibration parameters described above.

### Assessing the reliability of Dr. Diagnose

**Fig 5:**
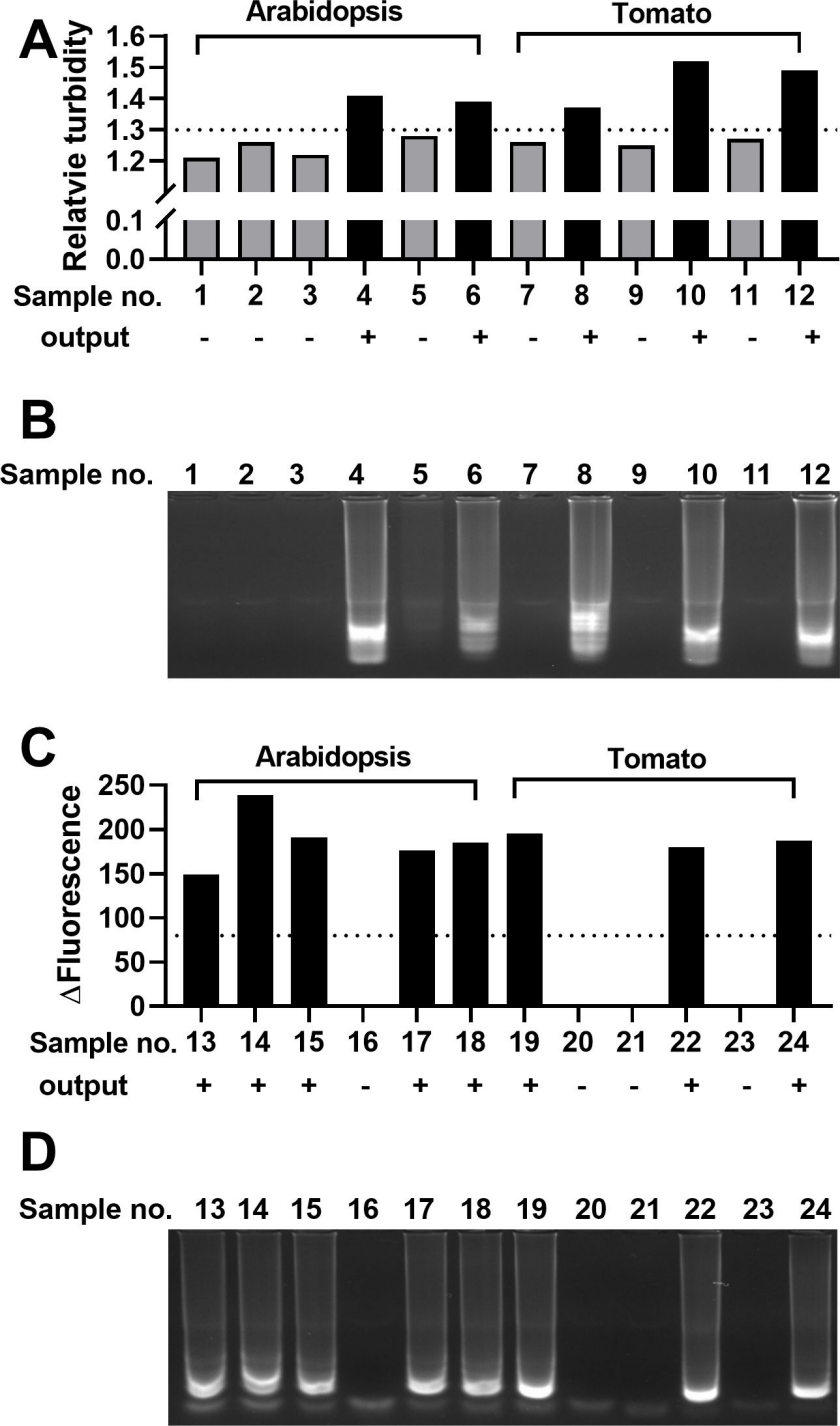
Reliability assessment. (**A**) *Arabidopsis thaliana* and tomato leaf samples were spiked with *Fusarium oxysporum* f. sp. *conglutinans* cultures to simulate infected plant samples, while non-spiked leaves were used as healthy samples. DNA from samples was purified using the dipstick method, and LAMP reactions were performed to detect *F. oxysporum*. The relative turbidity of each LAMP reaction measured by the device was calculated. The output interpreted by Dr. Diagnose for each sample is shown below each bar with “+” and “−” indicating positive and negative, respectively. (**B**) LAMP products from panel A were analyzed by agarose gel electrophoresis. (**C**) DNA from 12 biological samples with or without *F. oxysporum* was purified as described in panel A and eluted into LAMP reactions containing 2.5 µM SYTO9. The Δ Fluorescence values obtained by the Dr. Diagnose device were plotted, and the output result was shown below each bar. (**D**) LAMP products from panel C were analyzed by agarose gel electrophoresis.

To test the performance of the calibrated Dr. Diagnose, DNA was purified using the rapid dipstick DNA purification method (32) from *Arabidopsis thaliana* and tomato leaves with or without the addition of *Fusarium oxysporum* f. sp. *conglutinans* cultures to simulate infected and healthy plant samples, respectively. The purified DNA was eluted from the dipstick directly into LAMP reactions with primers designed to amplify *F. oxysporum* genomic DNA (Table 1). Dr. Diagnose was used to analyze the amplification reactions, and the results were verified using agarose gel electrophoresis. Analysis of 12 samples using turbidity measurement of Dr. Diagnose identified all five positive samples exceeding the threshold value of 1.3 (Fig. 5A), while gel electrophoresis confirmed these five samples displayed strong amplification (Fig. 5B). Sample number 5 had a relative turbidity of 1.28, just below the threshold value of 1.3, and displayed weak amplification barely visible by gel electrophoresis. A second set of 12 samples was analyzed using the same procedure, and the final amplification products were analyzed using the fluorescence readings of Dr. Diagnose (Fig. 5C and D). All eight samples showing amplification in the electrophoresis gel were correctly classified as positive, i.e., exceeding the threshold value of 80, by the device (Fig. 5C and D).

**TABLE 1:** Primers used

Name	Sequence (5′−3′)	Species	Source
LAMP-F3	TTGCGAACGTCACTTACCAA	*Fusarium oxysporum* f. sp. *conglutinans*	(11)
LAMP-B3	GAATATACCAATCTTGAGCAGAGCT		
LAMP-FIP	AGGGCCGCCGTTGAGATAGTCTTACCAACATCAGCAAGTATGG		
LAMP-BIP	GGAAAGCCCACCAACGGAGTTGTACCAGTGACCTTGATGAAC		
LAMP-F3	CTTATGAGTTGGACCCCCATT	Potato leafroll virus	Our study
LAMP-B3	TCCTGCGGGATCTGAAGA		
LAMP-FIP	TAAGTTTTGGCGCCGCCCTTCGCAAAGTATCATCCCTCCAGTC		
LAMP-BIP	AAGCGCGGATGATAAACGGGGCCATTTCCCTTCCACAGTATCC		
LAMP-F3	TTGTTGGCAACGGGAAGC	Tomato spotted wilt virus	(29)
LAMP-B3	AGGATCAGTTATAGTCCCCTG		
LAMP-FIP	GGCAGACCCATATCACAATCCTGAAGGTCATCAAGATCTGTCCA		
LAMP-BIP	AGGAAAACTTGTGGTTGCTCTGGCCCTTCAGAATGACTTGCTTT		
LAMP-Floop	TGTATTGTTTTCTGCTGTCCC		
LAMP-Bloop	CGATCCCAACATGCCATCTG		
LAMP-F3	TTGCAGTCGGTTTCGTCG	*Pseudomonas syringae* pv. tomato DC3000	(33)
LAMP-B3	GCGCAGCGCTGGATTG		
LAMP-FIP	TTGCCCGTCGGGTTAGCCGGGGTACACCGGTCGACAAT		
LAMP-BIP	AATCAGGACCTGGGTCAACTGCTGTTGCCAGCATCCTGAAGC		
PCR-F1	GAAAAGATGACCCAGATCAT	*Theobroma cacao*	Our study
PCR-R1	CCAGAATCCAGTACAATACC		
PCR-F2	CTATTGGAGCTGAGAGATTC		
PCR-R2	GCACAATGTTACCATAGAGA		

**Fig 6:**
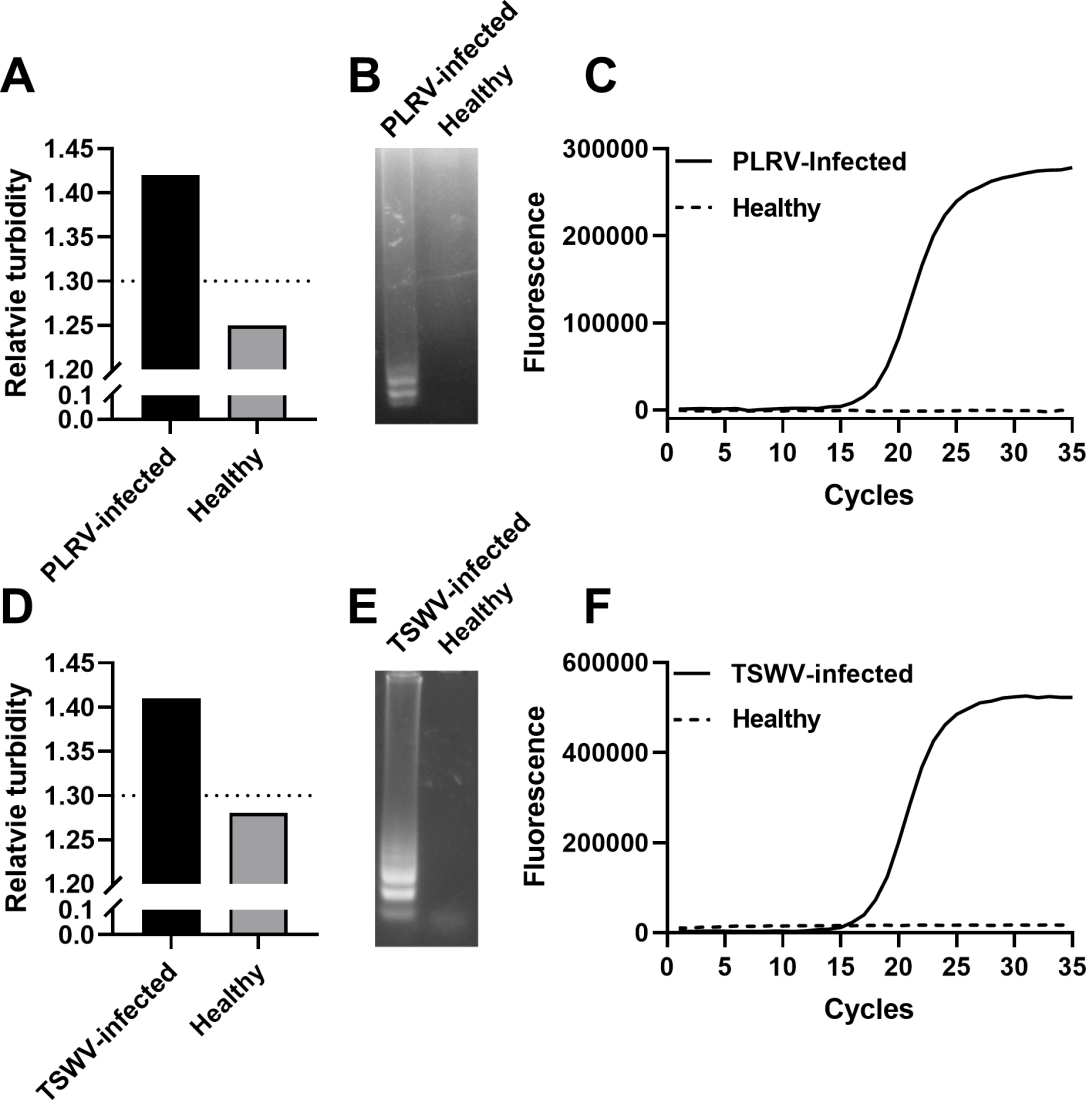
RNA virus detection. (**A**) By using the dipstick method, RNA from potato leafroll virus (RLRV)-infected or healthy *Physalis floridana* was purified and eluted into the LAMP reaction. After LAMP amplification was performed, Dr. Diagnose was used to measure the relative turbidity of each LAMP reaction. (**B**) LAMP products from panel A were analyzed by agarose gel electrophoresis. (**C**) To verify LAMP results, RNA from RLRV-infected or healthy *Physalis floridana* was extracted using the commercial RNA extraction kit and then reverse transcribed into cDNA. cDNA was used as the template in real-time PCR to identify the existence of RLRV. (**D**) By using the same procedure, Dr. Diagnose identified TSMV-infected and healthy tobacco. The results were verified using agarose gel electrophoresis (**E**) and real-time PCR (**F**).

Dr. Diagnose was also used to detect RNA viruses. *Physalis floridana* plants were infected with potato leafroll virus (PLRV). RNA was extracted from infected and control plants using dipstick technology and then eluted into LAMP reactions with specific PLRV primers (Table 1). Our results show that the relative turbidity value of the PLRV-infected sample exceeded the threshold of 1.3 and thus was identified as positive by Dr. Diagnose, while the control, healthy sample, was identified as negative by Dr. Diagnose (Fig. 6A). These results were verified by agarose gel electrophoresis and real-time PCR (Fig. 6B and C). Using a similar approach, RNA from the tobacco plant infected with tomato spotted wilt virus (TSWV) was purified using dipstick technology and then used as the template in LAMP amplification. Dr. Diagnose successfully identified TSWV-infected and healthy plants (Fig. 5D through F).

### Field demonstration of Dr. Diagnose

In the field, variable light conditions and temperature can interfere with PON diagnostics. We, therefore, evaluated the reliability of Dr. Diagnose in different conditions. In the case of Dr. Diagnose, the lid avoids interference from external light sources (Fig. 3C), and it successfully identified positive and negative samples based on their relative turbidity regardless of the light condition (Fig. S4A and B). In contrast, it was difficult to distinguish positive (cloudy) and negative (clear) samples using the naked eye unless it was placed against a black background (Fig. S4C). Additionally, Dr. Diagnose accurately identified positive and negative samples at different environmental temperatures including 5°C, 15°C, 20°C, 30°C, and 37°C (Fig. S5). Assays were also performed outside the laboratory to test the device in real field situations (Fig. S6A through F). For this purpose, we used a combination of dipstick technology, LAMP amplification, and turbidity-based Dr. Diagnose (Fig. S6A through F) to successfully detect *Pseudomonas syringae* pv. tomato DC3000 in infected *Arabidopsis thaliana*. Healthy *Arabidopsis thaliana* was identified as negative by Dr. Diagnose. The Dr. Diagnose results were confirmed by agarose gel and real-time PCR (Fig. S6G through I).

### PCR detection using Dr. Diagnose

Most DNA amplification technologies can be combined with fluorescence measurements. We thus tested whether Dr. Diagnose can be used to analyze the results of another amplification method (i.e., PCR). Three independent sets of PCR reactions containing SYTO9 were run for 0, 15, 20, 25, 30, 35, and 40 cycles and their amplification products were analyzed by the Dr. Diagnose device and gel electrophoresis. The Δ Fluorescence measured by Dr. Diagnose exceeded the value of 50 at 25 cycles, corresponding with a weak but observable band in the electrophoresis gel (Fig. 6A and B). The amount of amplicon observed by gel electrophoresis kept increasing after 30, 35, and 40 cycles in parallel with the Δ Fluorescence values measured by the device. The Δ Fluorescence value of 50 was then set as a threshold for PCR amplification, which was different from the threshold value of 80 for LAMP amplification. Dr. Diagnose was then reprogrammed to let the user choose PCR or LAMP mode before analysis (Movie S1).

To assess the reliability of Dr. Diagnose in PCR amplification, 71 PCR reactions producing either 119 or 150 bp amplicons were assessed with Dr. Diagnose. The device showed 100% accuracy in diagnosing PCR reactions displaying visible bands on agarose gels as positive, while reactions not showing a visible amplicon in the gel were correctly diagnosed as negative (Fig. 6C).

## DISCUSSION

Isothermal amplification technology is powerful in PON applications due to its single-temperature incubation requirement and the availability of a variety of naked-eye readout assays (34). However, naked eye-based readouts can be influenced by variable light conditions (30) or human bias, which may result in incorrect interpretation of the data (35). Here, we described the development of Dr. Diagnose, a device that allows the interpretation of LAMP and PCR amplifications in a rapid, reliable, and unbiased manner as a low-cost and easy-to-use replacement for naked-eye readout methods in PON applications.

Dr. Diagnose has a number of advantages over naked-eye readout methods. The device provides an unbiased interpretation of the data independent of the operator or the environment (e.g., light quality/intensity and temperature) in which the assay is performed (Fig. S4 and S5). Additionally, Dr. Diagnose was designed to analyze LAMP reactions without the requirement to open tubes post-amplification, which helps reduce false positives due to aerosol contamination (35). In contrast, some naked-eye assays need to add additional reagents or transfer amplicons post-amplification, and the opening of completed amplification reactions increases the risk of cross-contamination (6, 23).

Dr. Diagnose is an end-point detection device that provides the user with a number of advantages. First, it is easy to operate and does not need highly trained personnel. By simply touching a button, the interpreted results (i.e., the sample is positive/negative) are provided within seconds. Second, it does not require much electricity to run. The low (40 mA) power demand of the device allows it to be powered by a lightweight portable USB power bank supporting low-current charging typically used as mobile phone chargers. Third, our device provides users with the option to select the best option for isothermal incubators (e.g., heated water, exothermic chemical heaters) considering the sample size, cost, and accessible resources on site. The combination of the device and alternative heat sources (8, 15, 18) are suitable for remote field sites where there is no available electricity supply (Fig. S6). Fourth, the device is cheaper to produce. Dr. Diagnose has only 13 electronic components, three 3D-printed parts, and costs less than $5 USD. Fifth, as Dr. Diagnose has very few parts, the device has a small footprint (108 × 90 × 50 mm) and weighs less than 170 g, making it ideal for transport to remote areas.

Dr. Diagnose is a dual-function device, and its turbidity and fluorescence functions have different characteristics. Turbidity quantification identifies samples displaying clear and distinct LAMP banding patterns from those showing no or faint amplification on agarose gel analysis (Fig. 1 and 5), while fluorescence quantification provided additional sensitivity to identify weak amplifications (Fig. 2 and 5). Even though turbidity-based detection of LAMP reactions is less sensitive than fluorescence (36, 37), the use of fluorescent dyes increases costs, and the dyes need to be stored in the dark to avoid degradation. The dual functions of Dr. Diagnose provide users with an option to select a suitable detection method according to the purpose, budget, and accessible resources at PON.

**Fig 7:**
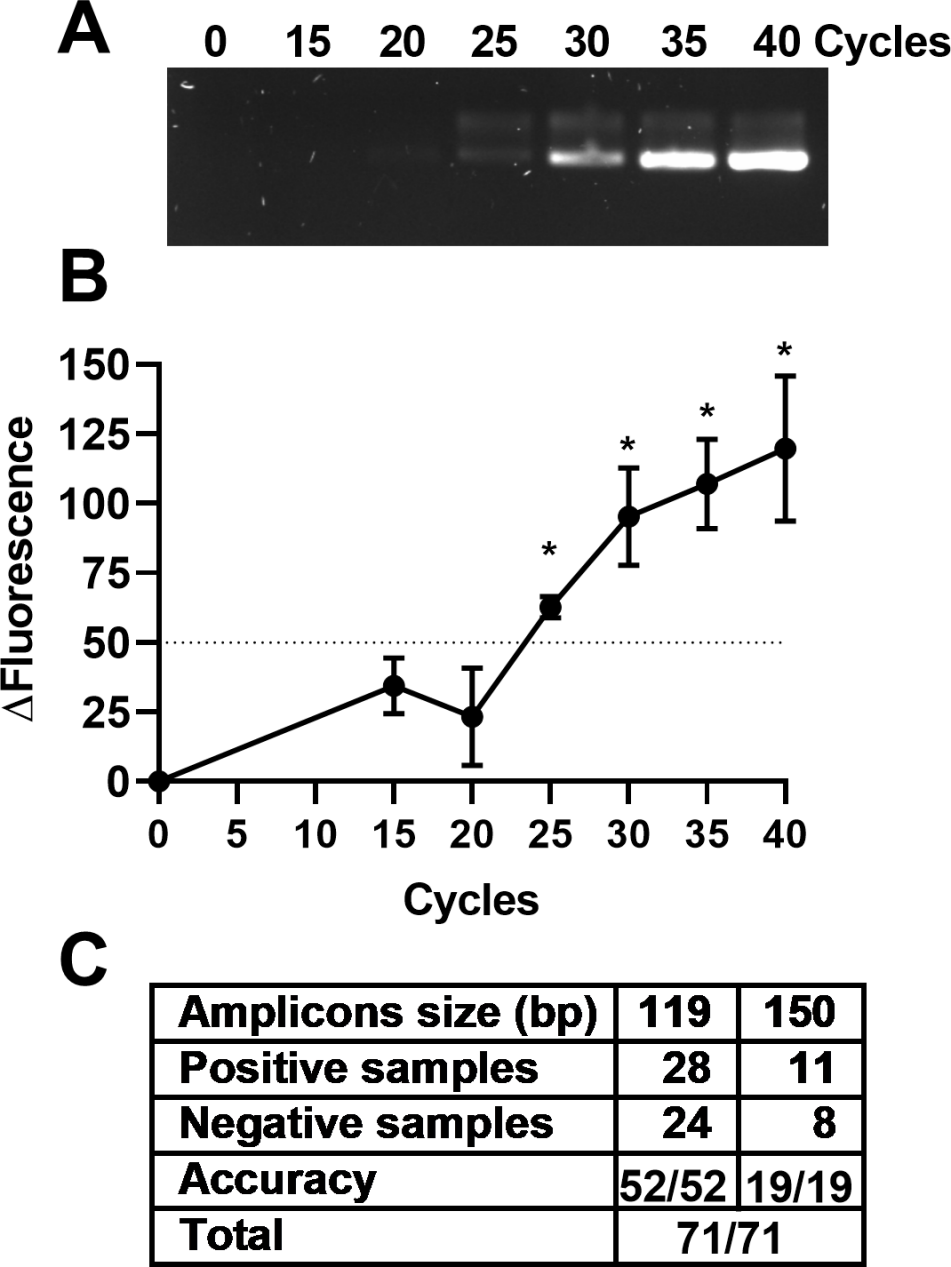
Dr. Diagnose application in PCR. (**A**) Identical PCR reactions were incubated for 0, 15, 20, 25, 30, 35, and 40 cycles before being visualized on an agarose gel. The picture shows the result of one out of three independent experiments with identical results. (**B**) Dr. Diagnose was used to measure Δ Fluorescence in each reaction using the respective starting (0 cycle) sample as control. (**C**) Two sets of PCR primers were used to amplify either 119 or 150 bp amplicons. A total of 71 PCR reactions were prepared and diagnosed as positive or negative by the device. All 71 reactions were analyzed by gel electrophoresis to determine the presence or absence of an amplicon.

Fluorescence measurements have been combined with multiple amplification methods for detection (6, 38). To test the compatibility of fluorescence measurement on Dr. Diagnose, the capability of Dr. Diagnose has been extended to evaluate the fluorescence of PCR amplification as an example (Fig. 7) due to the popularity of PCR-based assays in diagnostics and the portable commercially available PCR device (miniPCR, https://www.minipcr.com/products/minipcr/). After the proper threshold for PCR amplification was determined, the device distinguished between positive and negative samples among 71 samples with 100% agreement with traditional agarose gel electrophoresis (Fig. 7C). This reveals that the device can be used to detect amplification results of multiple methods as long as the suitable threshold is determined.

In summary, Dr. Diagnose is a reliable, inexpensive, and portable readout device for PON application. When used in combination with simple PON nucleic acid purification and isothermal amplification, Dr. Diagnose can form a complete sample-to-result system that enables diagnostics to be performed on-site.

## MATERIALS AND METHODS

### LAMP reaction parameters

Unless otherwise stated, each LAMP amplification reaction contained 0.8 M betaine, 8 mM MgSO_4_, 1.2 mM dNTP (each), 20 mM Tris-HCl (pH 8.8), 10 mM (NH_4_)_2_SO_4_, 10 mM KCl, 0.1% Triton X-100, 1.6 µM FIP primer, 1.6 µM BIP primer, 0.2 µM F3 primer, 0.2 µM B3 primer, and 0.32 U/µL Bst 2.0 warm start polymerase (New England Biolabs). Reactions were incubated at 63°C for 60 minutes followed by 5 minutes at 85°C to denature DNA polymerase and end amplification. Primers used in LAMP reactions are shown in Table 1.

### Concentration of LAMP amplicons

DNA concentration of LAMP amplicons was measured on a Quantus fluorimeter (Promega) using the QuantiFluor One dsDNA dye system following the manufacturer’s instructions. Briefly, 1 µL LAMP products or 1 µL 400 ng/µL QuantiFluor standard DNA was well mixed with 200 µL of QuantiFluor One dsDNA dye in 0.5 mL tube (Axygen, PCR-05-C) and incubated in the dark for 5 minutes to allow dsDNA dye bind to amplicons. The fluorimeter was calibrated using the standard before used to measure the concentration of each of the LAMP amplicons.

### Optimization of turbidity/fluorescence measurement

To obtain LAMP reactions with gradual cloudiness, 4 fg of LAMP amplicons was used as template DNA in a set of identical 50 µL LAMP reactions that were then incubated at 63°C for either 0, 5, 10, 15, 20, 25, 30, 35, 40, 45, 50, 55, or 60 minutes and then 5 minutes at 85°C. LAMP reactions post-amplification were checked using agarose gel electrophoresis and their respective DNA concentration was measured on a Quantus fluorimeter. The LAMP reactions were then used for either turbidity or fluorescence measurements. For fluorescence measurements, unless otherwise stated, the dsDNA intercalating dye SYTO9 (Invitrogen) was added to the LAMP reaction to a final concentration of 2.5 µM.

A light-emitting diode with a 450 nm wavelength peak (Supernatural, China) was used as a light source in both turbidity and fluorescence measurements. A phototransistor light sensor (Vishay TEPT4400) was set at either 90° or 180° to the LED light source (Fig. 1A). Unless otherwise stated, fluorescence measurements were made using a yellow photography lighting filter (eBay, http://mtw.so/6rzjOk) that was placed in front of the light sensor to block as much of the blue source light as possible while allowing the fluorescence to pass through. An Arduino UNO microcontroller was used to measure the turbidity or fluorescence of each sample that was placed between the sensor and the source light.

Unless otherwise stated, turbidity measurements were made by measuring the amount of light that is able to pass through the sample, with the sensor and light source in line with each other (i.e., 180° apart) (Fig. 3B). Thus, the relative turbidity of each sample was calculated by dividing light measurement of the blank (in the absence of a reaction tube) by the measured light data of the sample. For fluorescence measurements, the sensor and light source were placed perpendicular to each other (Fig. 3B), and the delta fluorescence of each sample was calculated by subtracting the light measurement of the negative control from that of the sample. At the initial stage of optimization of the fluorescence measurement, four technological replications were developed to generate data in Fig. S2A and B. All other data were obtained from two or three biological replications.

### Construction of a readout device

Light sensors for fluorescence and turbidity measurements were placed at 90° and 180° to the blue LED, respectively (Fig. 3B). Electronic components were soldered to a prototyping board and connected to an Arduino Pro Mini microcontroller as described in the circuit shown in Fig. 3E. To accommodate all electronic components, the case was designed using the online computer-aided design software Onshape (https://www.onshape.com/) (Fig. 3C), and the respective printer-specific g-code was generated by Ultimaker Cura (version 3.4.1). Polylactic acid and acrylonitrile butadiene styrene filament were used in the printing of the gray and black parts in Fig. 3C, respectively, on a 3D printer (Cocoon create touch) to obtain 3D-printed elements. The LED and light sensors were press-fitted into the 3D-printed sample holder (Fig. 3A and B), which was then glued in place in the body of the device. A sample holder lid was also printed so that the sample being analyzed could be covered to avoid stray light sources affecting measurements. Capacitive touch sensors were glued underneath the “T” or “F” symbol of the device’s top plate to enable the user to select between turbidity or fluorescence measurements. The main switch was placed at the side of the device body to control on/off of the power supply that could be provided by the portable 5 V USB battery by connecting it to the USB plug of the device. LCD was placed on top of the device to give instructions for operation and output interpreted results for the users.

### Device assessment

One 5 mm diameter leaf disc of *Arabidopsis thaliana* or tomato was added into a 2 mL screw cap tube containing one ball bearing and 500 µL extraction buffer (20 mM Tris pH 8, 25 mM NaCl, 2.5 mM EDTA, and 0.05% SDS). *Fusarium oxysporum* f. sp. *conglutinans* culture was spiked into plant leaf extract to simulate infected plant samples. The leaf sample was macerated by shaking tubes for 20 seconds, releasing the plant cell contents into the extraction buffer. A dipstick with a 2 × 6 mm^2^ nucleic acid binding area was then dipped into the leaf extract five times. DNA bound to the dipstick was washed by dipping the dipstick into 500 µL of 10 mM Tris (pH8) five times, and the DNA was eluted from the dipstick by dipping 15 times directly into a 30 µL LAMP reaction. For fluorescence measurements, the LAMP reaction contained 2.5 mM SYTO9. LAMP reactions were incubated at 63°C for 60 minutes and then at 85°C for 5 minutes. A total of 25 µL of LAMP products were used for the turbidity/fluorescence measurement on the device. Another 5 µL of LAMP product was examined using agarose gel electrophoresis to verify the results of the device.

To extract RNA from diseased and healthy *Physalis floridana* and/or tobacco, approximately 5 × 5 mm leaf sample was added into 1.5 mL tube containing 200 µL of extraction buffer (800 mM guanidine hydrochloride, 50 mM Tris, 0.5% Triton X-100, and 1% Tween 20). The disposable plastic pestle was then used to well grind the leaf sample. By using dipstick technology described above, RNA was eluted into LAMP reaction containing 8 mM MgSO_4_, 1.2 mM dNTP (each), 20 mM Tris-HCl (pH 8.8), 10 mM (NH_4_)_2_SO_4_, 150 mM KCl, 0.1% Triton X-100, 1.6 µM FIP primer, 1.6 µM BIP primer, 0.8 µM loop primer(s), 0.2 µM F3 primer, 0.2 µM B3 primer, and 0.32 U/µL Bst 3.0 DNA polymerase (New England Biolabs) with increased reverse transcriptase activity. Reactions were then incubated at 68°C (for PLRV detection) or 71.5°C (for TSWV detection) for 45 minutes and then at 85°C for 5 minutes.

### Real-time PCR

To identify RNA virus using real-time PCR, RNA was purified from plant samples using OminiPlant RNA Kit (DNaseI) (CWIBIO) and reverse transcribed into cDNA using ABScript III RT Master Mix for qPCR with gDNA Remover (ABclonal) as described by the manufacturer. cDNA was then added into real-time PCR reactions prepared using Genious 2× STBR Green Fast qPCR Mix (ABclonal). For DNA-based detection, DNA was purified using dipstick technology and then eluted into real-time PCR reactions. LAMP primers F3 and B3 were used in real-time PCR (Table 1).

### Field demonstration

*Pseudomonas syringae* pv. tomato DC3000-infected or healthy *Arabidopsis thaliana* leaf was added into a 1.5 mL tube containing 500 µL of extraction buffer (20 mM Tris pH 8, 25 mM NaCl, 2.5 mM EDTA, and 0.05% SDS). The disposable plastic pestle was then used to grind the leaf sample. By using dipstick technology, DNA was extracted and eluted into a pre-prepared LAMP reaction. Reactions were then incubated in a water bath provided by a travel cup for 60 minutes at the set temperature of 65°C. Dr. Diagnose was then used to identify each LAMP reaction.

### Statistics

Data sets containing only two groups were analyzed using unpaired *t*-test (nonparametric) in GraphPad Prism (*P* ≤ 0.05). All other data sets were analyzed using one-way ANOVA on Prism with a *post hoc* Tukey comparison of multiple means test (*P* ≤ 0.05).

## SUPPLEMENTAL MATERIAL

Fig. S1 to S6 aem.00902-23-s0001.docx Supplemental figures.

Movie S1 aem.00902-23-s0002.mp4 PCR mode of Dr. Diagnose.

ASM does not own the copyrights to Supplemental Material that may be linked to, or accessed through, an article. The authors have granted ASM a non-exclusive, world-wide license to publish the Supplemental Material files. Please contact the corresponding author directly for reuse.

## References

[1] Lau HY, Botella JR. 2017. Advanced DNA-based point-of-care diagnostic methods for plant diseases detection. Front Plant Sci 8:2016. doi:10.3389/fpls.2017.02016 29375588 PMC5770625

[2] Botella JR. 2022. Point-of-care DNA amplification for disease diagnosis and management. Annu Rev Phytopathol 60:1–20. doi:10.1146/annurev-phyto-021621-115027 36027938

[3] Donoso A, Valenzuela S. 2018. In‐field molecular diagnosis of plant pathogens: recent trends and future perspectives. Plant Pathology 67:1451–1461. doi:10.1111/ppa.12859

[4] Buja I, Sabella E, Monteduro AG, Chiriacò MS, De Bellis L, Luvisi A, Maruccio G. 2021. Advances in plant disease detection and monitoring: from traditional assays to in-field diagnostics. Sensors (Basel) 21:2129. doi:10.3390/s21062129 33803614 PMC8003093

[5] Hwang H, Bae S-C, Lee S, Lee Y-H, Chang A. 2013. A rapid and simple genotyping method for various plants by direct-PCR. Plant Breed Biotech 1:290–297. doi:10.9787/PBB.2013.1.3.290

[6] Duan Z, Yang X, Ji X, Chen Y, Niu X, Guo A, Zhu JK, Li F, Lang Z, Zhao H. 2022. Cas12a‐based on‐site, rapid detection of genetically modified crops. J Integr Plant Biol 64:1856–1859. doi:10.1111/jipb.13342 35962717

[7] Zou Y, Mason MG, Wang Y, Wee E, Turni C, Blackall PJ, Trau M, Botella JR. 2017. Nucleic acid purification from plants, animals and microbes in under 30 seconds. PLoS Biol 15:e2003916. doi:10.1371/journal.pbio.2003916 29161268 PMC5697807

[8] Mason MG, Blackall PJ, Botella JR, Templeton JM. 2020. An easy‐to‐perform, culture‐free campylobacter point‐of‐management assay for processing plant applications. J Appl Microbiol 128:620–629. doi:10.1111/jam.14509 31705613 PMC7027919

[9] Aula OP, McManus DP, Mason MG, Botella JR, Gordon CA. 2021. Rapid parasite detection utilizing a DNA dipstick. Exp Parasitol 224:108098. doi:10.1016/j.exppara.2021.108098 33713659

[10] Zhao Y, Chen F, Li Q, Wang L, Fan C. 2015. Isothermal amplification of nucleic acids. Chem Rev 115:12491–12545. doi:10.1021/acs.chemrev.5b00428 26551336

[11] Zou Y, Mason MG, Botella JR. 2020. Evaluation and improvement of isothermal amplification methods for point-of-need plant disease diagnostics. PLoS One 15:e0235216. doi:10.1371/journal.pone.0235216 32598374 PMC7323990

[12] Shi C, Shen X, Niu S, Ma C. 2015. Innate reverse transcriptase activity of DNA polymerase for isothermal RNA direct detection. J Am Chem Soc 137:13804–13806. doi:10.1021/jacs.5b08144 26474356

[13] Liao S-C, Peng J, Mauk MG, Awasthi S, Song J, Friedman H, Bau HH, Liu C. 2016. Smart cup: a minimally-instrumented, smartphone-based point-of-care molecular diagnostic device. Sens Actuators B Chem 229:232–238. doi:10.1016/j.snb.2016.01.073 26900258 PMC4756427

[14] Velders AH, Ossendrijver M, Keijser BJF, Saggiomo V. 2022. T-cup: a cheap, rapid, and simple home device for isothermal nucleic acid amplification. Glob Chall 6:2100078. doi:10.1002/gch2.202100078 35284091 PMC8902289

[15] LaBarre P, Hawkins KR, Gerlach J, Wilmoth J, Beddoe A, Singleton J, Boyle D, Weigl B. 2011. A simple, inexpensive device for nucleic acid amplification without electricity-toward instrument-free molecular diagnostics in low-resource settings. PLoS One 6:e19738. doi:10.1371/journal.pone.0019738 21573065 PMC3090398

[16] Hatano B, Maki T, Obara T, Fukumoto H, Hagisawa K, Matsushita Y, Okutani A, Bazartseren B, Inoue S, Sata T, Katano H. 2010. LAMP using a disposable pocket warmer for anthrax detection, a highly mobile and reliable method for anti-bioterrorism. Jpn J Infect Dis 63:36–40. doi:10.7883/yoken.63.36 20093760

[17] Ablordey A, Amissah DA, Aboagye IF, Hatano B, Yamazaki T, Sata T, Ishikawa K, Katano H. 2012. Detection of Mycobacterium ulcerans by the loop mediated isothermal amplification method. PLoS Negl Trop Dis 6:e1590. doi:10.1371/journal.pntd.0001590 22509415 PMC3317900

[18] Nkouawa A, Sako Y, Li T, Chen X, Nakao M, Yanagida T, Okamoto M, Giraudoux P, Raoul F, Nakaya K, Xiao N, Qiu J, Qiu D, Craig PS, Ito A. 2012. A loop-mediated isothermal amplification method for a differential identification of Taenia tapeworms from human: application to a field survey. Parasitol Int 61:723–725. doi:10.1016/j.parint.2012.06.001 22698671

[19] Yager P, Domingo GJ, Gerdes J. 2008. Point-of-care diagnostics for global health. Annu Rev Biomed Eng 10:107–144. doi:10.1146/annurev.bioeng.10.061807.160524 18358075

[20] Roskos K, Hickerson AI, Lu H-W, Ferguson TM, Shinde DN, Klaue Y, Niemz A. 2013. Simple system for isothermal DNA amplification coupled to lateral flow detection. PLoS One 8:e69355. doi:10.1371/journal.pone.0069355 23922706 PMC3724848

[21] Zhu X, Wang X, Han L, Chen T, Wang L, Li H, Li S, He L, Fu X, Chen S, Xing M, Chen H, Wang Y. 2020. Multiplex reverse transcription loop-mediated isothermal amplification combined with nanoparticle-based lateral flow biosensor for the diagnosis of COVID-19. Biosens Bioelectron 166:112437. doi:10.1016/j.bios.2020.112437 32692666 PMC7361114

[22] Goto M, Honda E, Ogura A, Nomoto A, Hanaki K-I. 2009. Colorimetric detection of loop-mediated isothermal amplification reaction by using hydroxy naphthol blue. Biotechniques 46:167–172. doi:10.2144/000113072 19317660

[23] Fischbach J, Xander NC, Frohme M, Glökler JF. 2015. Shining a light on LAMP assays' A comparison of LAMP visualization methods including the novel use of berberine. Biotechniques 58:189–194. doi:10.2144/000114275 25861931

[24] Zhang J, Hou Q, Ma W, Chen D, Zhang W, Wubshet AK, Ding Y, Li M, Li Q, Chen J, Dai J, Wu G, Zhang Z, Zaberezhny AD, Pejsak Z, Tarasiuk K, Zafar Khan MU, Wang Y, He J, Liu Y. 2022. A naked-eye visual reverse transcription loop-mediated isothermal amplification with sharp color changes for potential pen-side test of foot-and-mouth disease virus. Viruses 14:1982. doi:10.3390/v14091982 36146788 PMC9504329

[25] Cornelissen JHC, Sibma F, Van Logtestijn RSP, Broekman RA, Thompson K. 2011. Leaf pH as a plant trait: species‐driven rather than soil‐driven variation. Funct Ecol 25:449–455. doi:10.1111/j.1365-2435.2010.01765.x

[26] Mason MG, Botella JR. 2019. A simple, robust and equipment-free DNA amplification readout in less than 30 seconds. RSC Adv 9:24440–24450. doi:10.1039/c9ra04725e 35527854 PMC9069613

[27] Wee EJH, Lau HY, Botella JR, Trau M. 2015. Re-purposing bridging flocculation for on-site, rapid, qualitative DNA detection in resource-poor settings. Chem Commun 51:5828–5831. doi:10.1039/c4cc10068a 25622026

[28] Notomi T, Okayama H, Masubuchi H, Yonekawa T, Watanabe K, Amino N, Hase T. 2000. Loop-mediated isothermal amplification of DNA. Nucleic Acids Res 28:E63. doi:10.1093/nar/28.12.e63 10871386 PMC102748

[29] Fukuta S, Ohishi K, Yoshida K, Mizukami Y, Ishida A, Kanbe M. 2004. Development of immunocapture reverse transcription loop-mediated isothermal amplification for the detection of tomato spotted wilt virus from chrysanthemum. J Virol Methods 121:49–55. doi:10.1016/j.jviromet.2004.05.016 15350732

[30] Le TH, Nguyen NTB, Truong NH, De NV. 2012. Development of mitochondrial loop-mediated isothermal amplification for detection of the small liver fluke Opisthorchis viverrini (Opisthorchiidae; Trematoda; Platyhelminthes). J Clin Microbiol 50:1178–1184. doi:10.1128/JCM.06277-11 22322346 PMC3318558

[31] Vasilescu G. 2005. Electronic noise and interfering signals: principles and applications. Springer Science & Business Media.

[32] Mason MG, Botella JR. 2020. Rapid (30-second), equipment-free purification of nucleic acids using easy-to-make dipsticks. Nat Protoc 15:3663–3677. doi:10.1038/s41596-020-0392-7 33005038 PMC7528719

[33] Chen Z-D, Kang H-J, Chai A-L, Shi Y-X, Xie X-W, Li L, Li B-J. 2020. Development of a loop-mediated isothermal amplification (LAMP) assay for rapid detection of Pseudomonas syringae pv. tomato in planta. Eur J Plant Pathol 156:739–750. doi:10.1007/s10658-019-01923-8

[34] Bhat AI, Aman R, Mahfouz M. 2022. Onsite detection of plant viruses using isothermal amplification assays. Plant Biotechnol J 20:1859–1873. doi:10.1111/pbi.13871 35689490 PMC9491455

[35] Tao Z-Y, Zhou H-Y, Xia H, Xu S, Zhu H-W, Culleton RL, Han E-T, Lu F, Fang Q, Gu Y-P, Liu Y-B, Zhu G-D, Wang W-M, Li J-L, Cao J, Gao Q. 2011. Adaptation of a visualized loop-mediated isothermal amplification technique for field detection of Plasmodium vivax infection. Parasit Vectors 4:115. doi:10.1186/1756-3305-4-115 21693031 PMC3127850

[36] Amin Almasi M. 2013. Development and application of loop-mediated isothermal amplification assay for rapid detection of Fusarium oxysporum f. sp. lycopersici. J Plant Pathol Microbiol 04. doi:10.4172/2157-7471.1000177

[37] Mori Y, Kitao M, Tomita N, Notomi T. 2004. Real-time turbidimetry of LAMP reaction for quantifying template DNA. J Biochem Biophys Methods 59:145–157. doi:10.1016/j.jbbm.2003.12.005 15163526

[38] Qian W, Lu Y, Meng Y, Ye Z, Wang L, Wang R, Zheng Q, Wu H, Wu J. 2018. Field detection of citrus huanglongbing associated with ‘candidatus liberibacter asiaticus’ by recombinese polymerase amplification within 15 min. J Agric Food Chem 66:5473–5480. doi:10.1021/acs.jafc.8b01015 29781618

