# Supplementary material for: A low-cost, portable, dual-function readout device for amplification-based point-of-need diagnostics
Source: Appl Environ Microbiol. 2023 Dec 4;89(12):e00902-23. doi: 10.1128/aem.00902-23 (PMC10734478; doi:10.1128/aem.00902-23)
Supplement: Fig. S1 to S6 — Supplemental figures. [file aem.00902-23-s0001.docx]

# Supplementary material


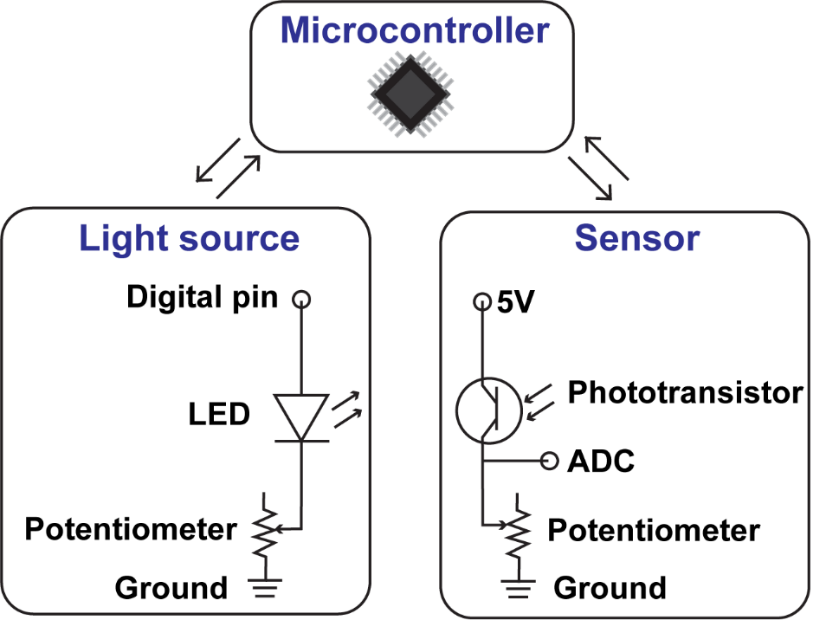


**Fig.S1.** Initial electronic circuit built for light measurement. LED, light emitting diode. ADC, Arduino’s analog-to-digital-convertor.


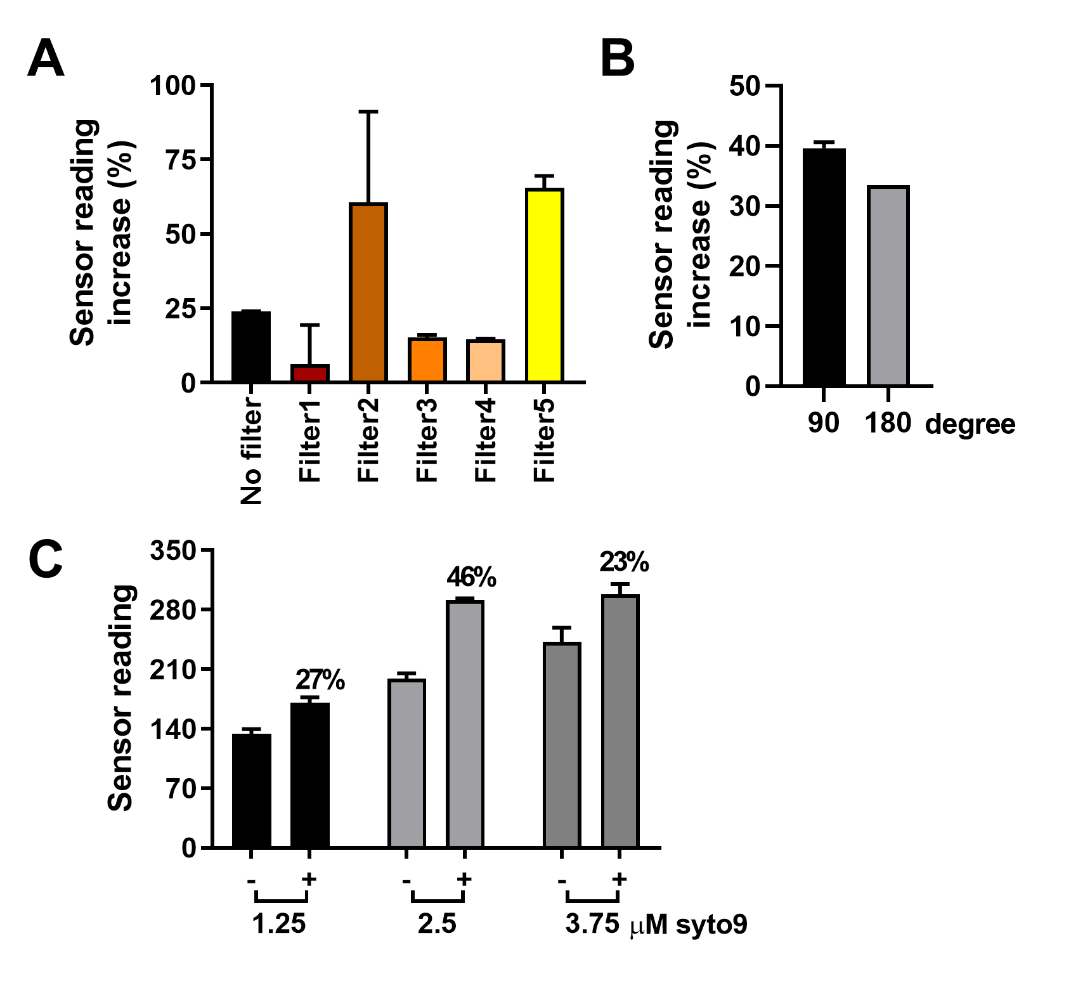


**Fig.S2.** Optimization of fluorescence measurements. (**A**) Positive and negative samples were measured by the light sensor using different color filters. The increases in readings between positive and negative reactions were calculated and plotted. The color of each filter has been used to fill its respective bar. (**B**) Reactions were measured by positioning the light sensor and a yellow filter either perpendicular (black) or in line (grey) with the LED. Increases in sensor readings between positive and negative reactions were calculated and plotted. (**C**) SYTO9 was added to negative (-) and positive (+) reactions to a final concentration of 1.25µM, 2.5µM and 3.75 µM. Completed reactions were measured, and readings were plotted in the bar graph. The number above each bar labeled with ‘+’ showed the average reading increase of positive vs negative samples.


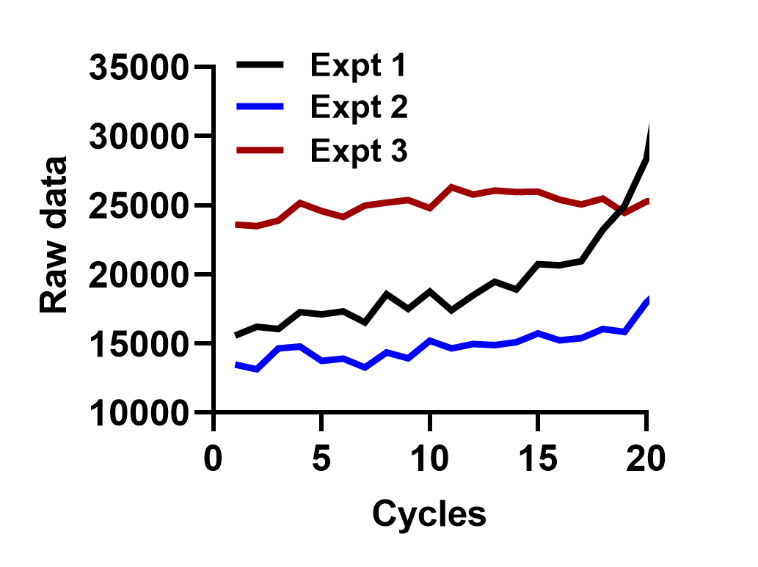


**Fig.S3.** Raw data of QuantStudio 1. Three independent quantitative real-time PCR was run in well A10 using SYBR mode of QuantStudio 1 (Applied Biosystems, USA). Raw data obtained by channel No.1 were plotted against cycles.


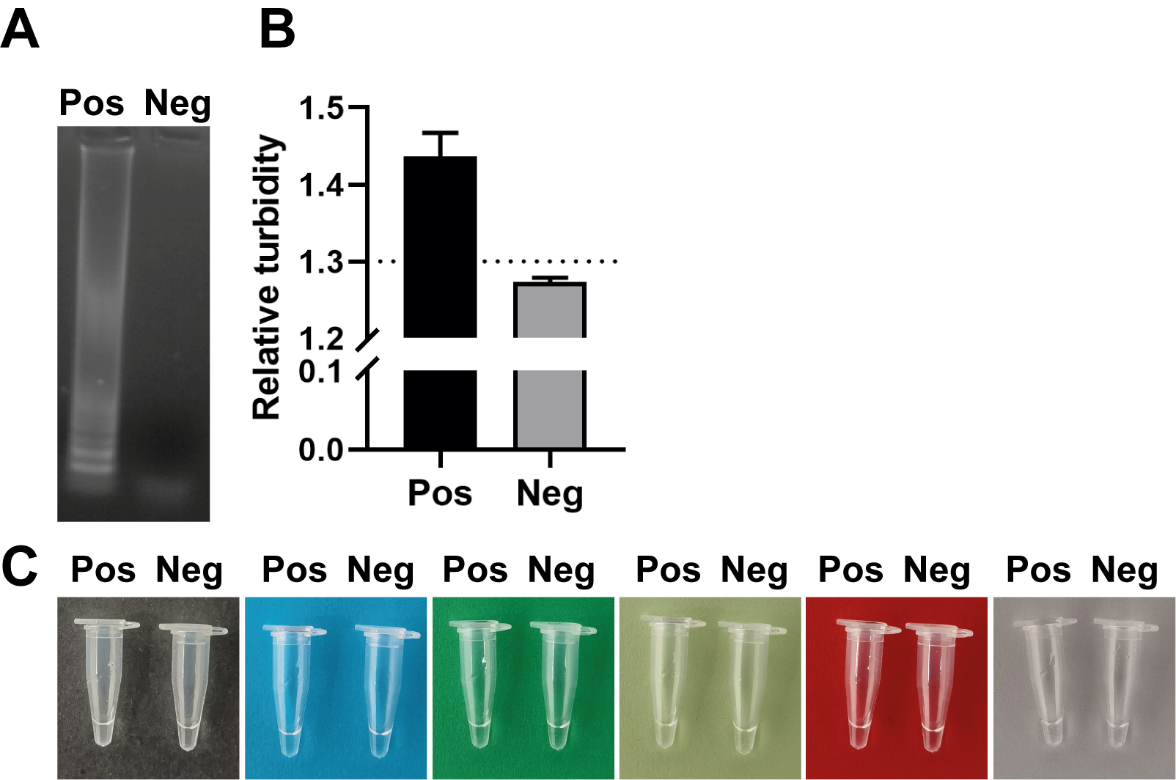


**Fig.S4.** Comparison between Dr. Diagnose and naked eyes. (**A**) Positive sample (Pos) displayed ladder-like pattern in agarose gel and negative sample (Neg) did not show visible amplicons. (**B**) Dr. Diagnose measured the relatively turbidity of each sample, and accurately identified positive and negative samples respectively according to the turbidity threshold of 1.3. Three technical replications were performed. (**C**) To simulate the performance of naked eyes in response to different light conditions, positive and negative samples were placed in black, blue, green, yellow, red and white backgrounds respectively.


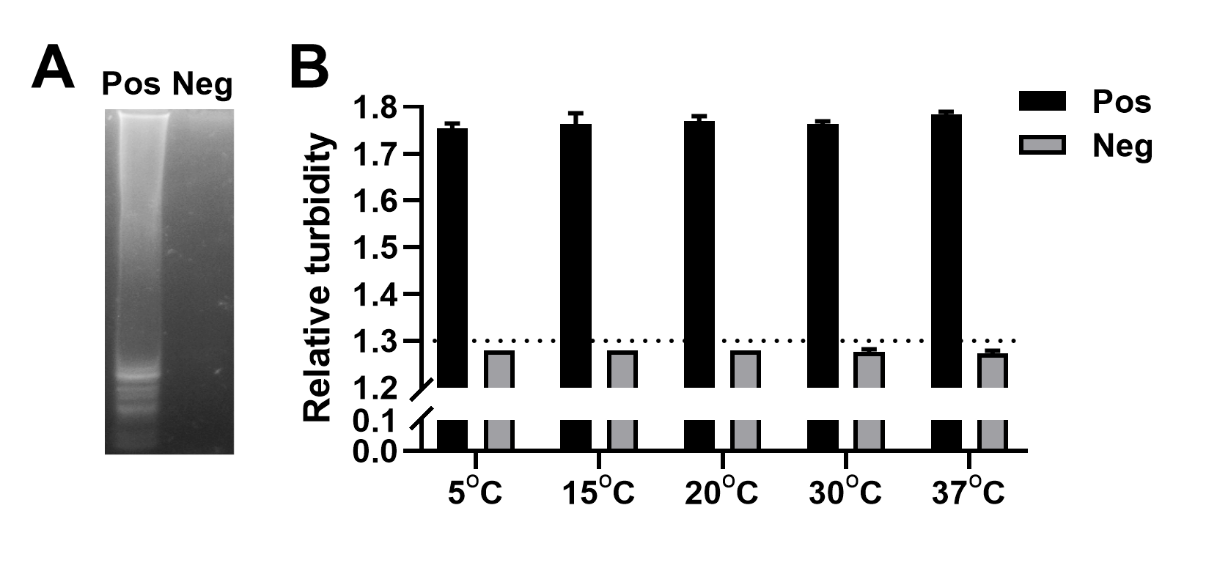


**Fig.S5.** Performance of Dr. diagnose at different temperatures. (**A**) Agarose electrophoresis assay was used to identify positive sample (Pos) and negative sample (Neg). (**B**) Dr. diagnose was placed at 5^o^C, 15^o^C, 20^o^C, 30^o^C and 37^o^C for at least 5 minutes and then used to identify each reaction by measuring relative turbidity. Three technical replications were performed.


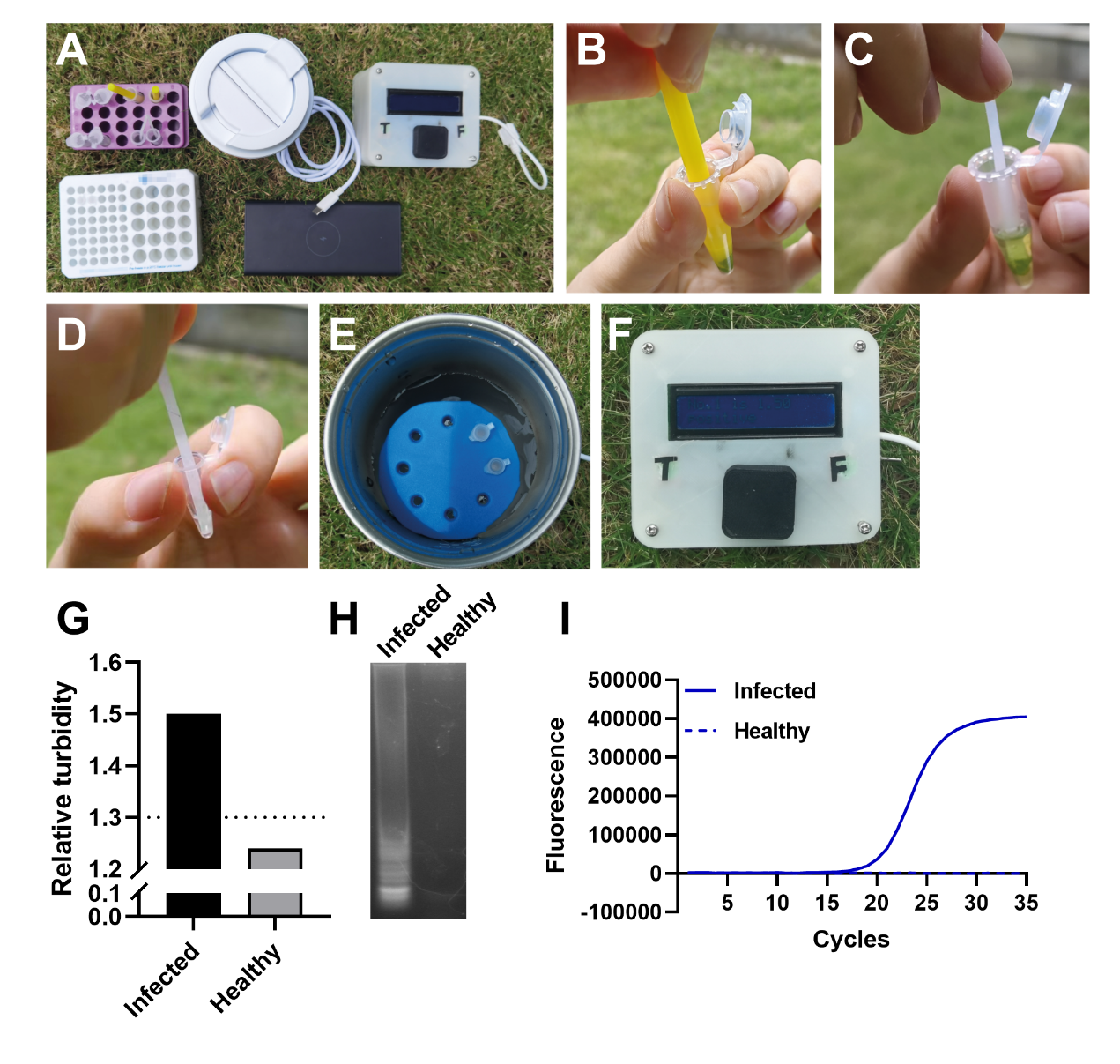


**Fig.S6.** Procedure of plant pathogen detection using Dr. Diagnose in the garden. (**A**) The diagnostics platform included DNA purification section, amplification section (i.e. pre-prepared LAMP reactions and travel cup) and read-out section (i.e. Dr. Diagnose). A portable power bank was used to power travel cup and Dr. Diagnose. (**B**) *Pseudomonas syringae*-infected or healthy leaf was added into 500ul DNA extraction buffer and ground using a deposable plastic pestle. (**C**) DNA was purified by using the dipstick method. (**D**) The purified DNA was eluted into LAMP reaction. (**E**) Reactions were incubated in trave cup powered by the portable power bank at a set temperature of 65^o^C for 60 minutes. (**F**) Dr. Diagnose was then used to evaluate samples. (**G**) Dr. Diagnose successfully identified infected and healthy samples by measuring the relatively turbidity. The result of Dr. Diagnose was verified by agarose electrophoresis assay (**H**) and real-time PCR assay (**I**).
